# Mesenchymal stem cell-derived exosomes ameliorate diabetic kidney disease through NOD2 signaling pathway

**DOI:** 10.1080/0886022X.2024.2381597

**Published:** 2024-07-22

**Authors:** Yinghui Wang, Donglin Lu, Shasha Lv, Xiangchun Liu, Gang Liu

**Affiliations:** a Department of Nephrology, Multidisciplinary Innovation Center for Nephrology, The Second Hospital, Cheeloo College of Medicine, Shandong University, Jinan, Shandong, China; b Nephrology Research Institute of Shandong University, Jinan, Shandong, China; c Key laboratory of Reproductive Endocrinology of Ministry of Education, Shandong University, Jinan, Shandong, China

**Keywords:** Diabetic kidney disease, exosomes, mesenchymal stem cells, NOD2

## Abstract

**Background and aims:**

Diabetic kidney disease (DKD) is one of the most common complications of diabetes. It is reported that mesenchymal stem cells (MSCs) derived exosomes (MSCs-Exo) may have great clinical application potential for the treatment of DKD, but the underlying mechanism has not been illustrated. To clarify the effect of MSC-Exo on NOD2 signaling pathway in podocytes under high glucose (HG) and DKD, we conduct this study.

**Methods:**

We co-cultured podocytes and MSCs-Exo under 30 mM HG and injected MSCs-Exo into DKD mice, then we detected the NOD2 signaling pathway by western blot, qRT-PCT, immunofluorescence, transmission electron microscopy and immunohistochemistry both *in vitro* and *in vivo*.

**Results:**

*In vitro*, HG lead to the apoptosis, increased the ROS level and activated the NOD2 signaling pathway in podocytes, while MSCs-Exo protected podocytes from injury reduced the expression of inflammatory factors including TNF-α, IL-6, IL-1β, and IL-18 and alleviated the inflammatory response, inhibited the activation of NOD2 signaling pathway and the expression of it’s downstream protein p-P65, p-RIP2, prevented apoptosis, increased cell viability in podocytes caused by HG. *In vivo*, MSCs-Exo alleviated renal injury in DKD mice, protected renal function, decreased urinary albumin excretion and inhibited the activation of NOD2 signaling pathway as well as the inflammation in renal tissue.

**Conclusion:**

MSCs-Exo protected the podocytes and DKD mice from inflammation by mediating NOD2 pathway, MSCs-Exo may provide a new target for the treatment of DKD.

## Introduction

According to the data of the International diabetes Federation, in 2021, there were 537 million adults suffered from diabetes mellitus (DM) in the whole world. It is estimated that by 2045, this number will increase to more than 780 million. Among them, it is estimated that 90–95% have type 2 diabetes (T2D) and early half of T2D patients will develop diabetic kidney disease (DKD) [1]. DKD is one of the most common microvascular complications of Diabetes (DM) and also one of the main causes of end-stage renal disease (ESRD) [2]. The main clinical manifestation of DKD is the progressive reduction of proteinuria and glomerular filtration rate (GFR) [3]. Clinical strategies to prevent the development of DKD mainly includes controlling blood glucose and blood pressure, dietary therapy, lipid-lowering therapy, end-stage renal replacement therapy or organ transplantation. There are many current treatment drugs for DKD. Clinical experiments revealed that renin-angiotensin-aldosterone system (RAS) inhibitors, especially angiotensin-converting-enzyme inhibitor (ACE-I) and angiotensin II receptor blocker (ARB), was found to be effective in treating DKD. However, although RAS inhibitors reduce proteinuria and slow the decline of glomerular filtration rate (GFR), they cannot suppress ESKD development completely. Besides, they could increase the incidence of many complications, such as AKI and hyperkalemia. Sodium-glucose cotransporter 2 (SGLT2) inhibitor such as dapagliflozin and engagliflozin, is the new strategies for DKD, especially the combination therapy with RAS and SGLT2 inhibitor is effective in controlling the progression of DKD [4]. However, there are many patients whose treatment effects cannot delay the progression of DKD to ESRD. Therefore, it is of great significance to explore the pathogenesis of DKD and seek new and more effective treatment methods.

Till now, the exact pathogenesis of DKD has not been fully elucidated [5]. DKD is usually classified as a noninflammatory glomerular disease; however, genome-wide transcriptome analysis studies consistently indicate the presence of inflammatory signaling pathways in the context of DKD [6]. Studies have revealed that innate immunity and inflammation plays significant roles in the progress of DKD [7]. As the important part of the innate immune system, NOD-like receptors (NLRs) are the intracellular sensors of microbes and danger signals, could identify the pathogen-associated molecular patterns (PAMPS) and damage-associated molecular patterns (DAMPs) which are released by damaged and dying cells, and promotes sterile inflammation [7]. Nucleotide-binding and oligomerization domain containing protein 2 (NOD2) is a cytosolic receptor belonging to the NLR family. Structurally, two tandem N-terminal caspase recruitment domain (CARDs) constitute the NOD2 protein and interact with downstream molecules which contain CARD as well. After activation by ligands, through CARD to CARD interaction, NOD2 recruit downstream receptor interacting protein 2 (RIP2), then the activated RIP2 leads to the phosphorylation of nuclear factor-kappa B inhibitor (IκB), resulting in the activation of nuclear factor-kappa B (NF-κB), which translocates to the nucleus and starts transcription of proinflammatory genes and eventually leading to the inflammation [8,9]. Several studies have showed that, in DKD, NOD2 is involved in the production and persistence of inflammation [10,11]. Targeting of NOD2 signaling pathway could be beneficial for the treatment of DKD.

Mesenchymal stem cells (MSCs) are multipotent cells with self-renewal, regenerative, proliferative, and multi-lineage differentiation potential and have shown promising results in experimental DKD [12,13]. However, due to its several disadvantages, such as safety, immune rejection and ethical problems, there are limitations for its application in clinical treatment. In addition to possess the function of MSCs, the adverse reactions of MSCs can be avoided to a certain extent by their extracellular vesicles (EVs), including exosomes with a diameter of 30–120 nm, and micro-vesicles ranging from 100 nm to 1 μm in size. Mounting evidence supports that MSCs released vesicles (MSCs-EVs), which may have greater clinical application potential than that of MSCs [14]. In recent years, MSC-Exo is becoming a novel and promising cell-free therapeutic agent in many ongoing clinical studies Several clinical research revealed that MSC-Exos could against different diseases, including kidney diseases, osteoarthritis, stroke, Alzheimer’s disease and type 1 diabetes [15–18].

In this study, we conducted CCK-8, western blot analysis, qRT-PCT, immunofluorescence, transmission electron microscopy and immunohistochemistry to investigate the mechanism of NOD2 in podocytes injury and DKD mice as well as the role of MSCs-EVs in this process. Our founds may be of great significance for the treatment of DKD.

## Materials and methods

### Cell culture

The immortalized human podocytes were provided by Dr. Fan Yi (Shandong University). Firstly, podocytes were proliferated in a 33 °C and 5% CO_2_ incubator, latter, they were transferred to a 37 °C and 5% CO_2_ incubator for at least 2 weeks for differentiation as described [19]. Then, they were cultured in RPMI 1640 medium supplemented with 10% FBS (Gibico, 10099-141c USA) and 1% penicillin/streptomycin (Solarbio, P1400, China). Medium contained 5.5 mM D-glucose as NG group; medium contained 30 mM D-glucose as HG group; medium contained 30 mM D-glucose and Exo(30 μg/mL) as Exo group; 24.5 mM D-mannitol was added in the medium as the osmotic control (Man group).

The extraction, culturation and identification of HUC-MSCs were conducted according to the guidelines of the Medical Ethics Committee as described [20].

### Exosome isolation and characterization and labeling

Exosome isolation kit (System Biosciences, EXOTC10A-1, USA) was used to isolate exosomes from conditional medium of the 4th passage of HUC-MSCs as described [21]. The protein concentration of exosomes were detected by a BCA Protein Assay Kit (Beyotime, P0009, China). Transmission electron microscopy (TEM) was used to observe the morphology of exosome. Nanosight tracking analysis (NTA) was used to analyze size distribution of exosomes in accordance with the manufacturers’ protocols. Western blot analysis were conducted to determine the specific protein markers of exosomes CD63, CD81 and TSG101.

HUC-MSCs derived exosomes were labeled with green fluorescent PKH67 dye (Sigma-Aldrich, PKH67GL, USA) following the manufactures’ protocols as described [22]. To observe the uptake of exosomes, *in vitro*, PKH67 labeled exosomes were incubated with podocytes for 12, 24 and 48 h and observed by confocal microscopy; *in vivo*, PKH67 labeled exosomes were injected into DKD mice and kidneys were harvested after 8 h and observed under the confocal microscopy.

## Transmission electron microscopy (TEM)

Electron microscopic sample handling and the TEM detection of exosomes and kidney tissue were performed by the electron microscopic core lab of Shandong Normal University. The analysis of TEM images including the foot process width (FPW), GBM thickness and the number of foot processes were calculated as described [23].

### Podocytes viability assay

A Cell Counting Kit-8 (CCK-8, Dojindo, CK04, Japan) was used to measure the viability of podocytes according to the manufacturer’s protocols. Podocytes were seeded into 96-well plates for 24h, then they were cultured with different concentration of glucose or AGEs, with or without exosomes. After 48 h of incubation, podocytes were incubated with 200 μl fresh medium and 10 μl CCK-8 at 37 °C.Cell viability was measured by a microplate reader at a wavelength of 450 nm and compared with the control groups [23]. Each experiment was repeated three times.

### Podocytes apoptosis

Podocytes were seeded into 6-well plates for 24h, then they were cultured with different concentration of glucose or AGEs, with or without exosomes. After 48 h of incubation, flow cytometric analysis of AnnexinV-PI double staining (Elabscience, E-CK-A211, China) was used to detected the apoptosis. After digested by trypsin and washed with cold PBS, podocytes were resuspended in 100 μl AnnexinV/PI binding buffer and incubated with 5 μl of AnnexinV-FITC and 5 μl of PI for 30 min at room temperature in the dark and then analyzed in flow cytometer within one hour. Viable cells were scored as those that were negative for AnnexinV and PI. The stained cells were analyzed by flow cytometry to determine the percentages of AnnexinV+/PI-(early apoptosis)and AnnexinV+/PI+(late apoptosis)cells [24]. The experiment was replicated for three times.

### Podocytes ROS

A fluorescent probe dihydroethidium (DHE, Beyotime, S0063, China), was used to detect intracellular ROS levels in podocytes. For DHE staining, podocytes were incubated with 10 μM DHE for 30 min at 37 °C and then observed by LSM780 laser scanning confocal microscope (Zeiss, Germany) [25].

### Immunofluorescence (IF)

Podocytes and renal cortex tissues were firstly fixed with 4% paraformaldehyde, podocytes were incubated with phalloidin (1:100; Sharebio, SB-YP0052, China), nephrin (1:100, Santa Cruz, sc-376522, China), NOD2 (1:200; Invitrogen, MA1-16611, USA); the renal tissues were incubated with anti-WT-1 (1:50, Abcam, ab89901, USA), nephrin (1:100, Santa Cruz, sc-376522, China) and synaptopodin (1:300; Sigma, ABN481, USA) antibodies overnight at 4 °C. Then podocytes and renal tissues were incubated with FITC-labeled/rhodamine-labeled secondary antibody (1:100, Zsbio, ZF-0311/ZF-0312, China) for 1 h at 37 °C. After washed with PBS, DAPI (1:1000, Solarbio, China) was used to counterstain the cell nuclei for 5 min [26]. Images were observed by a confocal microscopy (ZEN, Germany) and assessed by ImageJ software.

### Animal studies

Seven-week-old C57BL/6J male mice were obtained from GemPharmatech Co Ltd (Nanjing, China). After feeding with high-fat diet (HFD, 60.3 kcal% fat; 5.1 kcal/g, TD.09766, Teklad Custom Research Diets, Envigo) for 6 weeks to induce insulin resistance, newly prepared STZ solution was injected into mice intraperitoneally (100 mg/kg, for three days) to destroy pancreatic islets β cells, decrease the secretion of insulin, lead to an increase in blood glucose, while the control group was given an equal volume of PH4.48 citrate buffer solution. 72 h later, mice were considered diabetic if blood glucose exceeding 16.7 mmol/L. After feeding for 12 weeks, mice were considered DKD if urinary albumin was positive.

12-Week-old db/db and nondiabetic db/m control mice were obtained from GemPharmatech Co Ltd (Nanjing, China). The mice were housed in an SPF environment, the temperature was 22–24 °C and the humidity was 40–60%. All animal experiments were conducted following standards and procedures approved by the Animal Ethics Committee of Shandong University. Among the DKD mice, four of them were injected with MSCs-Exo (100 μg MSCs-Exo in 0.2 mL PBS 3 times in the first week, and then twice a week in the next 3 weeks); four of them were injected with insulin (Jiangsu, Wan bang Pharmaceutical, China); four of them were injected with PBS (0.2 mL). The specific treatment of mice, the detection of blood glucose and the harvest of kidneys have been previously described [23].

## Quantitative real-time polymerase chain reaction (qRT-PCR)

Total RNA was isolated from cells and renal tissues with Trizol reagent (Invitrogen, USA) and the mRNA levels of NOD2, nephrin were analyzed by real-time quantitative RT-PCR as described [23]. PCR included priming at 42 °C for 2 min; reverse transcription at 37 °C 15 min and 85 °C 5s; RT inactivation at 95 °C for 2 min, 95 °C for 5 min and 60 °C for 50s to cycle 40 times, melting curve stage; optimization step at 4 °C. *GAPDH* was used as controls for mRNA normalization and 2^−△△Ct^ method was used to calculate the relative expression of mRNA. The specific primers for *NOD2, nephrin* and *GAPDH w*as listed in Table 1.

**Table 1.** Specific primers for qRT-PCR.

Gene	Spices	Primer sequence(5′–3′)	Product size (bp)	Annealing (°C)	Cycle
*NOD2*	Human	F: CACCGTCTGGAATAAGGGTACT R: TTCATACTGGCTGACGAAACC	229	60.9 60	40
*NOD2*	Mouse	F: TGGACACAGTCTGGAACAAGG R: TGGACACAGTCTGGAACAAGG	103	61.6 60.3	40
*Nephrin*	Human	F: TCACCGTGAATGTTCTGTTCC R: AGTGTGGCTAAGGGATTACCC	133	60.2 60.9	40
*Nephrin*	Mouse	F: CAGCGATGATGCGGAGTACG R: CAGCTACCCAGGTAACTGTGC	149	63 62.4	40
*GAPDH*	Human	F: GGAGCGAGATCCCTCCAAAAT R: GGCTGTTGTCATACTTCTCATGG	197	61.6 60.9	40
*GAPDH*	Mouse	F: TGGCCTTCCGTGTTCCTAC R: GAGTTGCTGTTGAAGTCGCA	178	61.3 60.9	40

### ELISA

The levels of inflammatory factors in cell supernatant and renal tissue: TNF-α, IL-6, IL-1β, IL-18 were detected according to the instructions of the ELISA kits from Thermo Fisher Scientific for TNF-α(BMS2034/KMC3011), IL-6 (BMS213-2/900-K50), IL-1β(900-t95/EM2IL1B), IL-18(KHC0181/KMC0181).

### Western blot analysis

Total protein preparation and western blot analysis were conducted as described previously [23]. Anti-GAPDH (1:3000, TA-08, Zsbio, China), anti-NOD2 (1:1000, Invitrogen, MA1-16611, USA), anti-p-P65 (1:1000, Invitrogen, MA5-15160, USA), anti-p-RIP2 (1:1000, Invitrogen, PA5-118495, USA), anti-nephrin (1:500,Santa Cruz, sc-376522, China) (anti-nephrin was used for the characterization of podocytes *in vivo* and *in vitro*) were used as primary antibodies; housekeeping protein GAPDH was used as internal control and the results were analyzed by image J software.

### Immunohistochemistry (IHC) and histological observation of kidneys

IHC analysis of NOD2 was performed as we previously described [23]. Periodic acid Schiff (PAS) (Solarbio, G1281, China), masson staining (Solarbio, G1340, China) were conducted to assess glomerular sclerosis and kidney injury according to the manufacturer’s protocols.

### Statistical analysis

Data are expressed as means ± SD. Statistical analyses were performed with Student’s *t-*test for comparisons between two groups and one-way analysis of variance (ANOVA) for multiple comparisons. Statistical significance was accepted at values of *p* < 0.05.

## Results

### Identification and localization of HUC-MSCs derived exosomes

**Figure 1.**
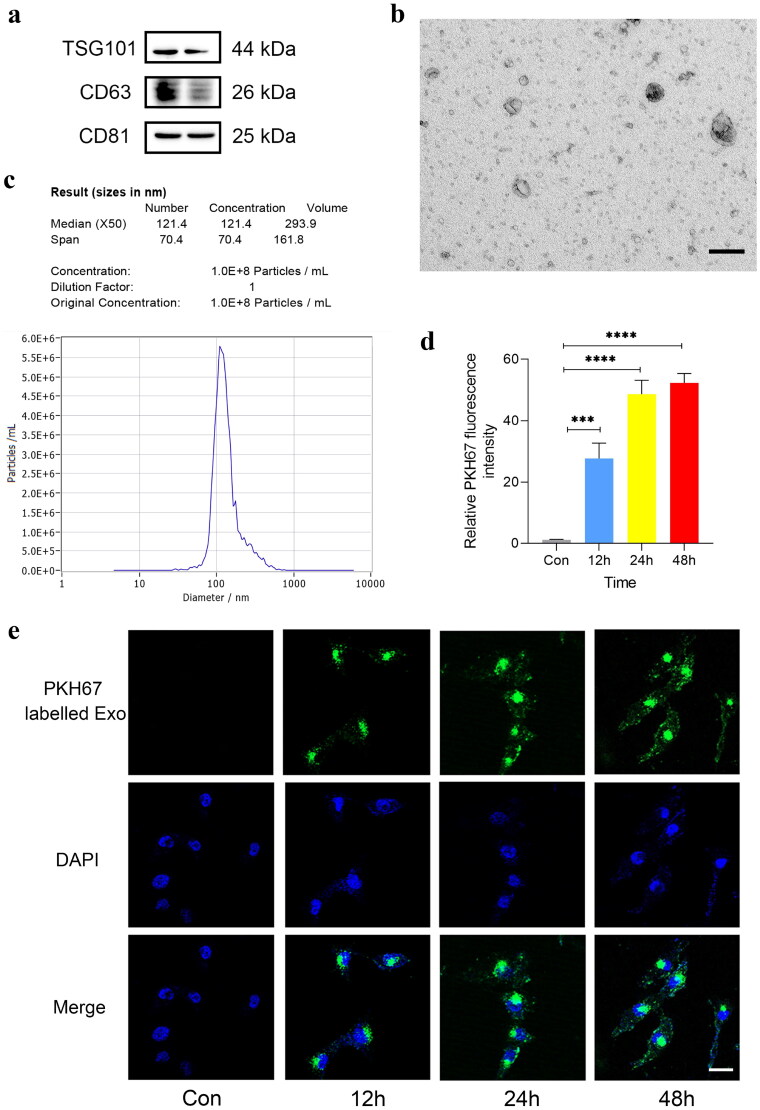
Identification and localization of HUC-MSCs derived exosomes. (a) Representative Western blot gel documents showing the protein expression of TSG101, CD63, CD81 in MSCs-Exo. (b) Morphology of MSCs-Exo under transmission electron microscopy (bar = 100μm). (c) Nanoparticle tracking method showing the diameters and concentrations of MSCs-Exo. (d,e) Representative immunofluorescent staining and summarized data showing the uptake of PKH67-labeled MSCs-Exo by podocytes at different time points (12, 24 and 48 h) (*n* = 3, bar = 10μm).

First of all, transmission electron microscopy (TEM), nanosight tracking analysis (NTA)and western blot was used to analyze the morphology and surface markers of exosomes that we extracted from the culture supernatants of MSCs. Western blot analysis indicated that exosomes possessed the exosomal surface marker proteins CD63, CD81, and exosomal luminal protein TSG101 (Figure 1(a). The morphology of exosomes under TEM was shown as Figure 1(b). Diameters and concentrations) of exosomes was shown as Figure 1(c). To observe the uptake of exosomes by podocytes, we used a confocal fluorescence microscopy to detect the immunofluorescence in podocytes after co-cultured with PKH67 labeled MSCs-Exo at 12, 24, 48h, the results showed that green fluorescence intensities was higher over time, indicating that PKH67 labeled MSCs-Exo was observed by podocytes (Figure 1(d,e)).

### MSCs-exo has protective effects on podocytes

**Figure 2.**
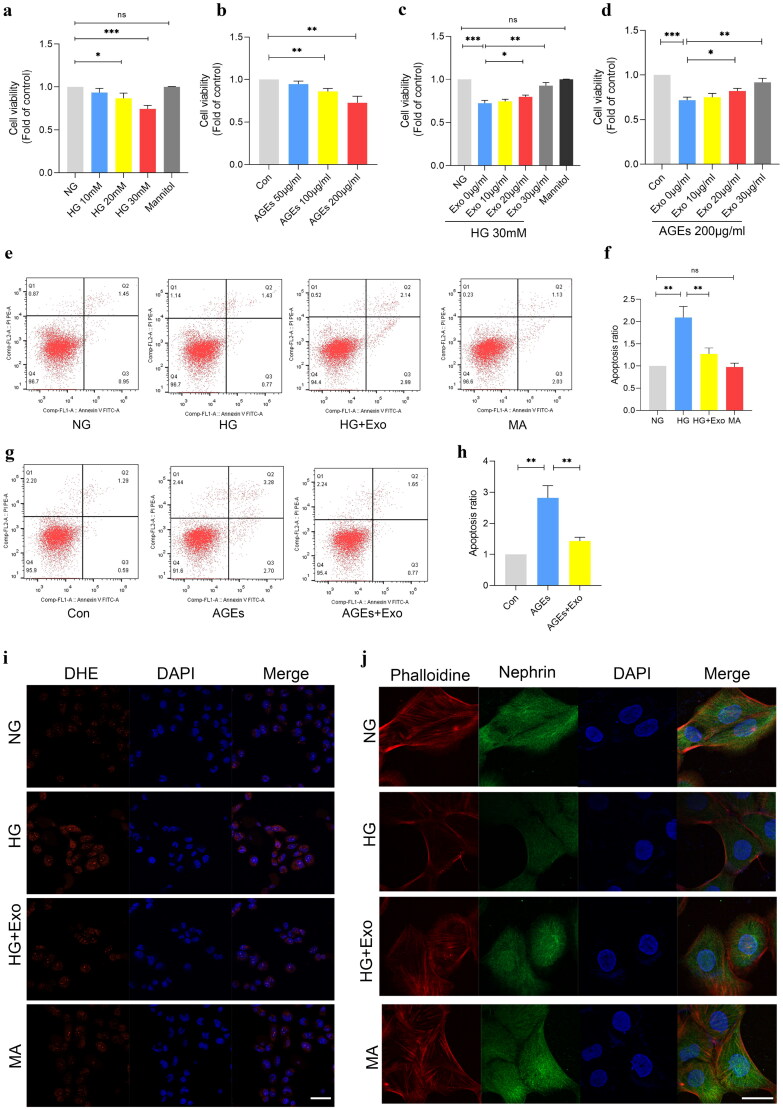
Protective effects of MSCs-exo on podocytes. (a,b) The viability of podocytes under different doses of HG and AGEs for 48 h (*n* = 3). (c,d) Respective viability of podocytes treated with increasing doses of MSCs-Exo under 30 mM HG and 200 μg/ml AGEs for 48 h (*n* = 3). (e,f) Summarized data showing podocyte apoptosis under HG (30 mM) with or without MSCs-Exo (30μg/ml) for 48 h determined by flow cytometric analysis (*n* = 3). (f,h) Summarized data showing podocyte apoptosis under 200 μg/ml AGEs with or without MSCs-Exo (30μg/ml) for 48 h determined by flow cytometric analysis (*n* = 3). (i) Representative images of ROS changes in podocytes under HG (30 mM) with or without MSCs-Exo (30μg/ml) for 48 h visualized by DHE (red) staining and nuclei (blue) staining (bar = 20μm). (j) Representative confocal microscopic images of F-actin using phalloidine (red) staining, nephrin (green) staining and nuclei (blue) staining in podocytes under HG (30 mM) with or without MSCs-Exo (30μg/ml) for 48 h (*n* = 3, bar = 20μm). Exo: HUC-MSCs derived exosomes; NG: normal glucose; HG: high glucose; AGEs: advanced glycation end products; Data are represented as mean ± SD, *p < 0.05; **p < 0.01; ***p < 0.001; *ns*: not significant.

To confirm MSCs-Exo can protect podocytes under common detrimental factors in DKD such as high glucose (HG) and AGEs, firstly, CCK-8 and flow cytometry were used to investigate the effects of MSCs-Exo on podocytes viability and apoptosis under HG and AGEs. As shown in Figure 2(a,b), HG and AGEs decreased the cell viability of podocytes dose-dependently, while MSCs-Exo increased the podocytes viability as concentrations increased under 30 mM HG and 200 μg/mL AGEs (Figure 2(c,d)). Similarly, podocytes apoptosis was increased under 30 mM HG and 200 μg/mL AGEs, while MSCs-Exo (both 30 μg/mL) suppressed this effect (Figure 2(e–h)). Moreover, immunofluorescence showed that, HG increased the ROS level of podocytes compared to the normal glucose and MSCs-Exo could decrease the ROS level of podocytes under 30 mM HG (Figure 2(i)). In addition, we further observed that MSCs-Exo improved podocytes cytoskeleton rearrangement under 30 mM HG (Figure 2(j)).

### NOD2 was highly expressed in podocytes under HG and DKD mice

**Figure 3.**
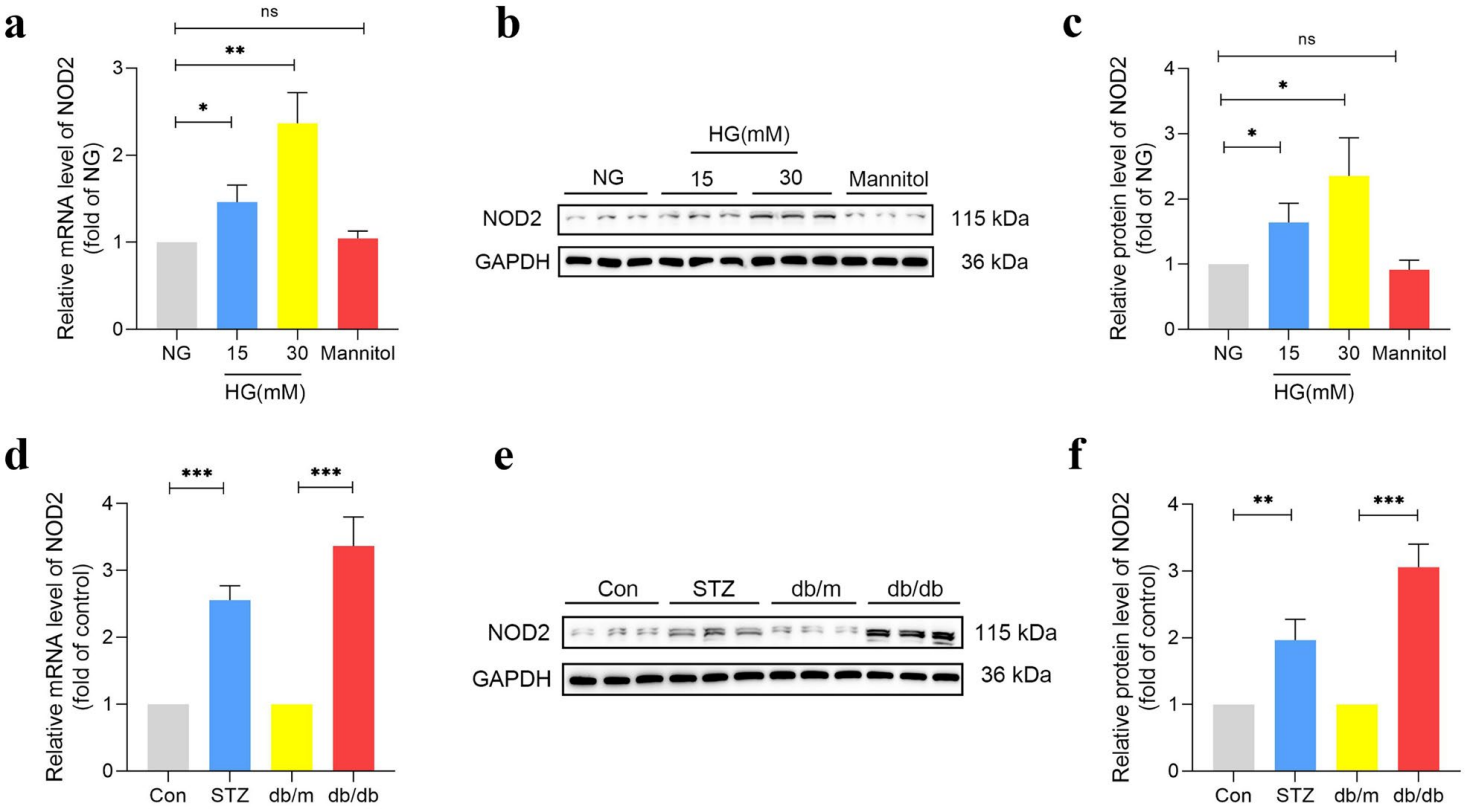
The expression of NOD2 in podocytes and DKD mice. (a) Relative mRNA level of NOD2 in podocytes under different concentrations of HG for 48 h (*n* = 3). (b, c) Representative western blot gel documents and summarized data showing the protein levels of NOD2 in podocytes under different concentrations of HG for 48 h (*n* = 3). (d) Relative mRNA levels of NOD2 in the kidney from HFD/STZ-induced DKD mice and db/db mice (*n* = 3). (e, f) Representative Western blot gel documents and summarized data showing the protein levels of NOD2 in the kidney from HFD/STZ-induced DKD mice and db/db mice (*n* = 3). NG: normal glucose; HG: high glucose; data are represented as mean ± SD, *p < 0.05; **p < 0.01; ***p < 0.001; *ns*: not significant.

Recent years, several studies have revealed that DKD is an inflammatory disease and NOD2 was highly expressed in DKD, to confirm that, RT-PCR and western blot were conducted to detect the expression of NOD2 in podocytes and DKD mice. Our results indicated that NOD2 was significantly increased in podocytes under 30 mM HG both in mRNA and protein level (Figure 3(a–c)). Then we detected the NOD2 expression in kidneys of DKD mice of normal control mice, we found that NOD2 was significantly increased in HFD/STZ induced DKD mice especially in db/db mice (Figure 3(d–e)). Therefore, our results demonstrated that NOD2 was highly expressed in DKD.

### MSCs-Exo inhibited the activation of NOD2 signaling decreased the inflammation of podocytes under HG

**Figure 4.**
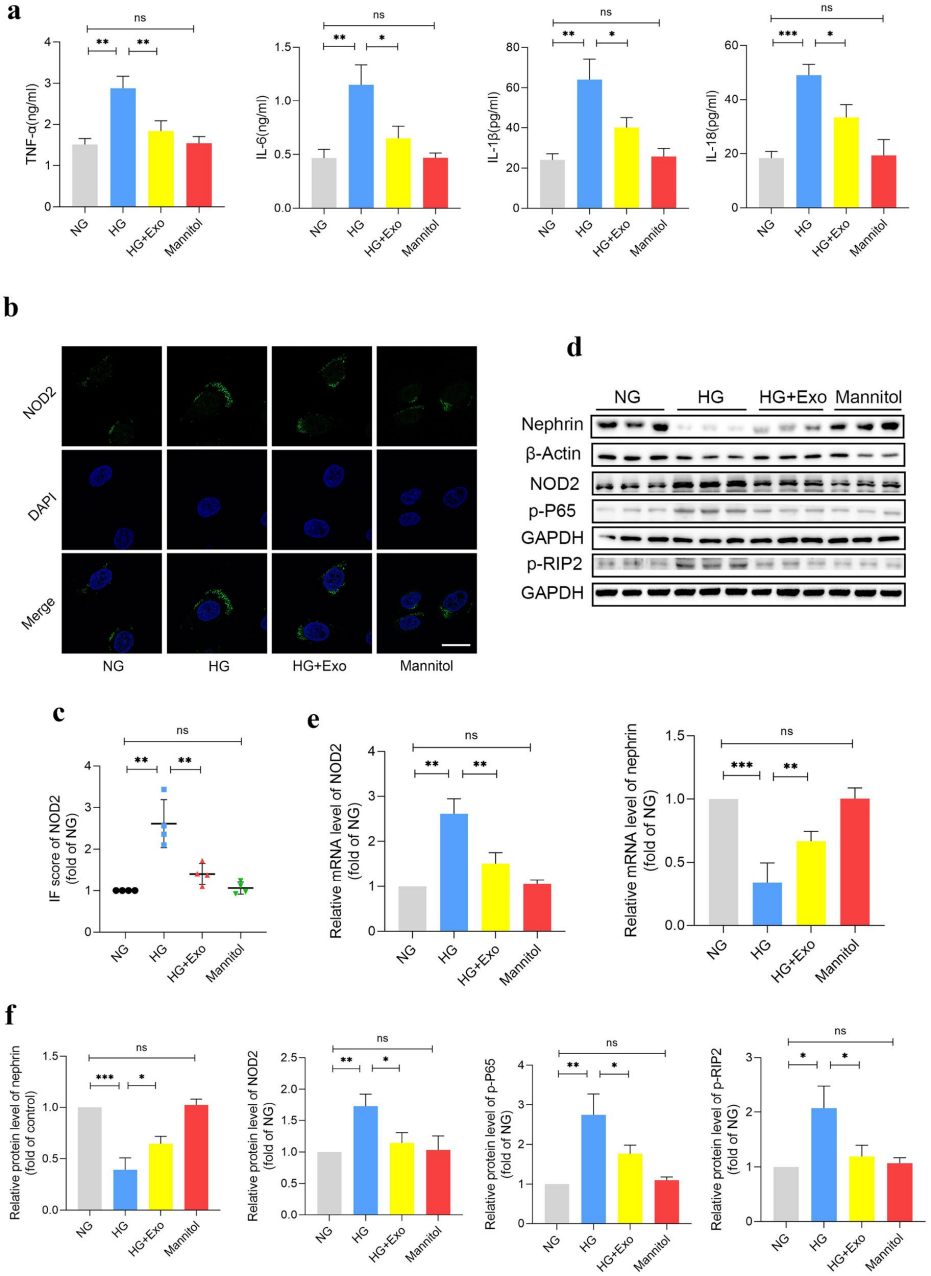
MSCs-Exo specifically inhibit NOD2 signaling activation in podocytes under HG. (a) Levels of proinflammatory factors (TNF-α, IL-6, IL-1β, and IL-18) expression in supernatants from podocytes with different treatments for 48 h (MSCs-Exo: 30μg/ml; HG: 30mM; *n* = 3). (b, c) Representative immunofluorescent staining and summarized data showing the effect of MSCs-Exo (30 μg/ml) on the expression of NOD2 in podocytes with different treatments for 48 h (MSCs-Exo: 30μg/ml; HG: 30mM for 48 h; *n* = 3; bar = 10μm). (e) Relative mRNA level of NOD2 and podocyte marker-nephrin in podocytes with different treatments for 48 h (MSCs-Exo: 30μg/ml; HG: 30mM for 48 h; *n* = 3). (d, f) Representative Western blot gel documents and summarized data showing the protein levels of nephrin, NOD2, p-P65 and p-RIP2 in podocytes with different treatments for 48 h (MSCs-Exo: 30μg/ml; HG: 30mM for 48 h; *n* = 3). NG: normal glucose; HG: high glucose; Exo: HUC-MSCs derived exosomes. Data are represented as mean ± SD; *p < 0.05; **p < 0.01; ***p < 0.001; *ns*: not significant.

Since we have demonstrated that NOD2 was highly expressed in DKD, we hypothesized that MSCs-Exo targets the NOD2 signaling to protect podocytes and DKD. To investigate this hypothesis, ELISA, RT-qPCR, western blot and immunofluorescence was conducted to detect the NOD2 signaling pathway in podocytes. After co-cultured with 30 μg/mL MSCs-Exo under 30 mM HG for 48h. Our results showed that HG activated the NOD2 signaling pathway, increased the inflammatory factors TNF-α, IL-6, IL-1β, and IL-18 in supernatants (Figure 4(a)), the mRNA and protein expression of NOD2 as well as the protein levels of NOD2 downstream signaling protein p-P65, p-RIP2(Figure 4(b–f)). Moreover, the podocyte marker nephrin was also significantly decreased under HG (Figure 4(d,e)). However, MSCs-Exo inhibited the activation of NOD2 signaling decreased the inflammation of podocytes under HG to a certain extent. Our results suggested that HG results in inflammation by activating the NOD2 signaling pathway in podocytes *in vitro* and that MSCs-Exo protects podocytes by preventing this progression.

### MSCs-exo ameliorated DKD mice

**Figure 5.**
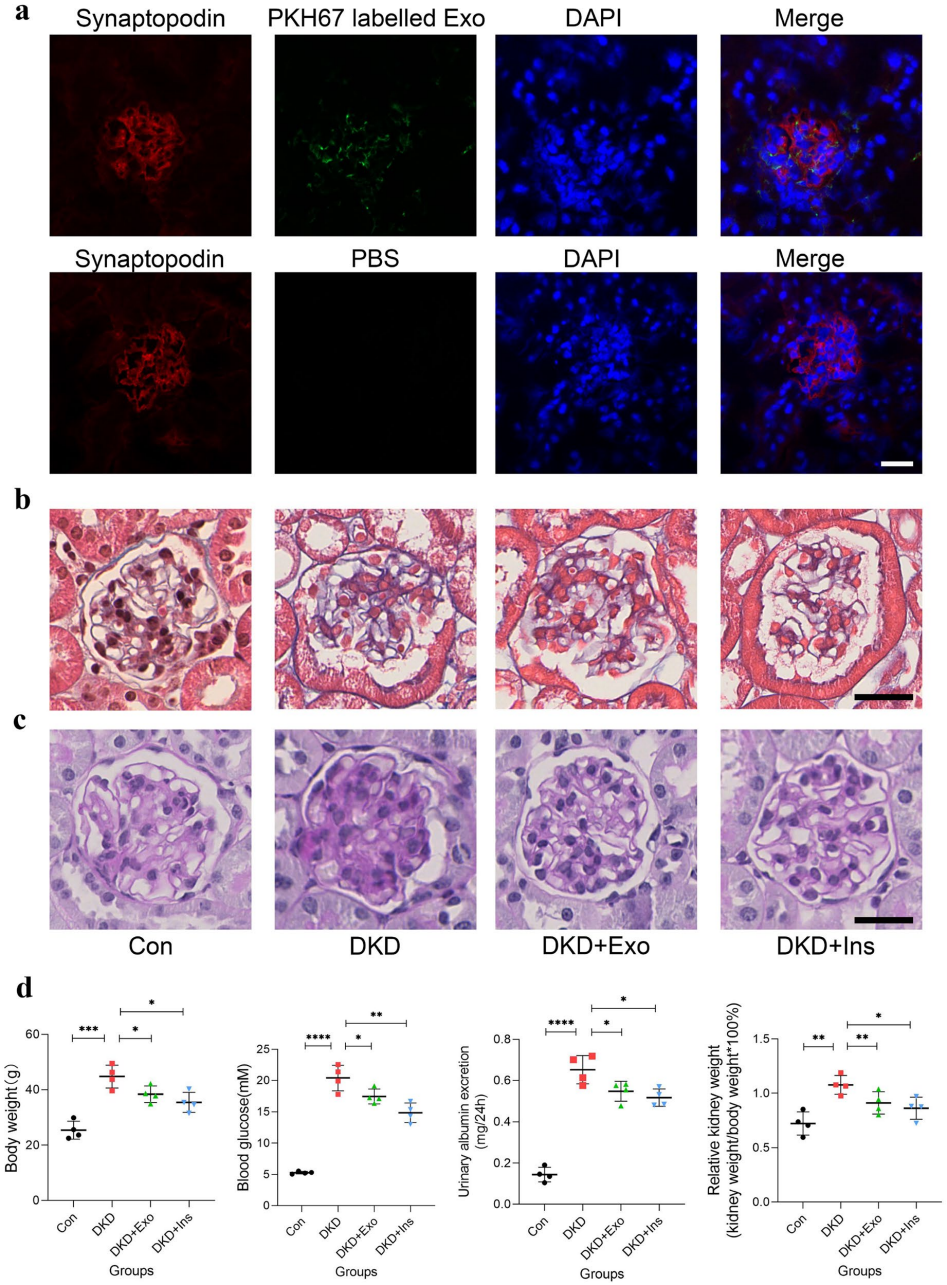
MSCs-Exo ameliorate renal injury in DKD mice. (a) Representative immunofluorescent staining showing the localization of PKH67-labeled Exo (*n* = 3, bar = 20μm). (b) Representative images of Masson staining showing the renal fibrosis in kidneys (*n* = 4, bar = 20μm). (c) Representative images of PAS staining showing the typical glomerular structure changes in kidneys (*n* = 4, bar = 20μm). (d) Physical and biochemical parameters of four groups of mice (*n* = 4). Exo: HUC-MSCs derived exosomes; Ins: insulin; Con: db/m mice; DKD: db/db mice; DKD + Exo: db/db mice with Exo; DKD + Ins: db/db mice with insulin. Data are represented as mean ± SD; *p < 0.05; **p < 0.01; ***p < 0.001; ^****^p < 0.0001.

Firstly, to confirm the MSCs-Exo could locate in glomerulus, we injected PKH67 labeled MSCs-Exo into DKD mice, 8h later we obtained the kidneys and tracked the PKH67 labeled MSCs-Exo by immunofluorescence, at the same time, PBS was injected into the other DKD mice as control, confocal microscope observed that green fluorescence was detected mostly in the glomerulus, however, there was no fluorescence in the glomerulus that was injected with PBS, indicating that MSCs-Exo localized to the DKD kidney (Figure 5(a)). Next, to explore the protection of MSCs-Exo in DKD, we injected MSCs-Exo and insulin into DKD mice, and db/m mice without diabetes were used as normal controls. After 8 weeks treatment, our results showed that, compared to the normal controls, DKD mice had higher body weight, blood glucose, urinary albumin excretion, and relative kidney weight. However, MSCs-Exo and insulin treatment relieved these indexes, especially the insulin treatment (Figure 5(d)). The range of weight for the 4 groups of mice was: 25.43 ± 3.245(Con); 44.75 ± 4.1753.245(DKD); 38.35 ± 2.963(DKD + Exo); 35.43 ± 3.6(DKD + Ins). Masson staining revealed renal tissue in red and the collagen fibers in blue in DKD group (Figure 5(b)) and PAS staining revealed a significant increase in glomerular glycogen deposition in the DKD group (Figure 5(c)), indicated that renal fibrosis and injury in DKD mice were more serious than the normal controls, but alleviated in the MSCs-Exo transplantation and insulin treatment groups compared with the DKD groups.

**Figure 6.**
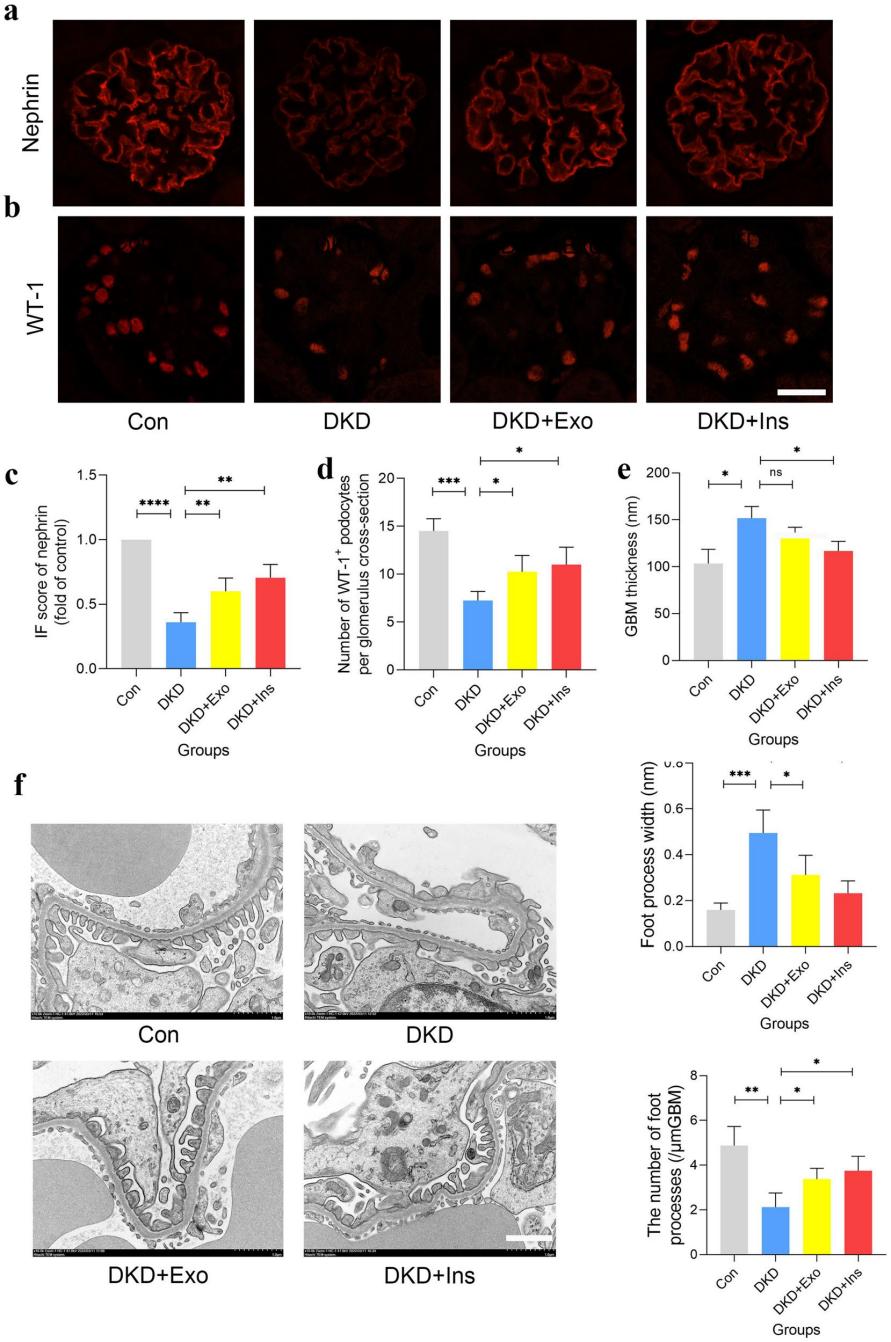
Protective effects of MSCs-exo in DKD mice. (a, c) Representative immunofluorescent staining and summarized data showing the expression of podocyte marker: nephrin in the kidney from different groups of mice (*n* = 4, bar = 20μm). (b, d) Representative immunofluorescent staining and summarized data showing the WT-1^+^ podocytes in the kidney from different groups of mice (*n* = 4, bar = 20μm). (e) Representative photomicrographs showing morphological changes in podocytes foot processes in different groups of mice by transmission electron microscopy (TEM) analyses (*n* = 4, bar = 1μm, 10000×). (f) Quantifications of mean glomerular basement membrane (GBM) thickness, mean foot process width, and the number of foot processes in different groups of mice by TEM analyses (*n* = 4). Exo: HUC-MSCs derived exosomes; Ins: insulin; Con: db/m mice; DKD: db/db mice; DKD + Exo: db/db mice with Exo; DKD + Ins: db/db mice with insulin. Data are represented as mean ± SD, *p < 0.05l **p < 0.01; ***p < 0.001; ^****^p < 0.0001; *ns*: not significant.

Moreover, immunofluorescence indicated that compared with controls, the levels of podocyte marker nephrin and Wilms Tumor-1^+^ (WT-1^+^, a specific marker for podocyte) was highly decreased in DKD mice compared to the other three groups (Figure 6(a–d)). Transmission electron microscopy (TEM) analyses further revealed the morphological changes and injuries in podocytes and glomerular basement membrane (GBM), including pronounced GBM thickening and podocyte foot process effacement in DKD mice which was improved by MSCs-Exo and insulin treatment (Figure 6(e,f)).

### MSCs-exo restrained DKD inflammation by mediating NOD2

**Figure 7.**
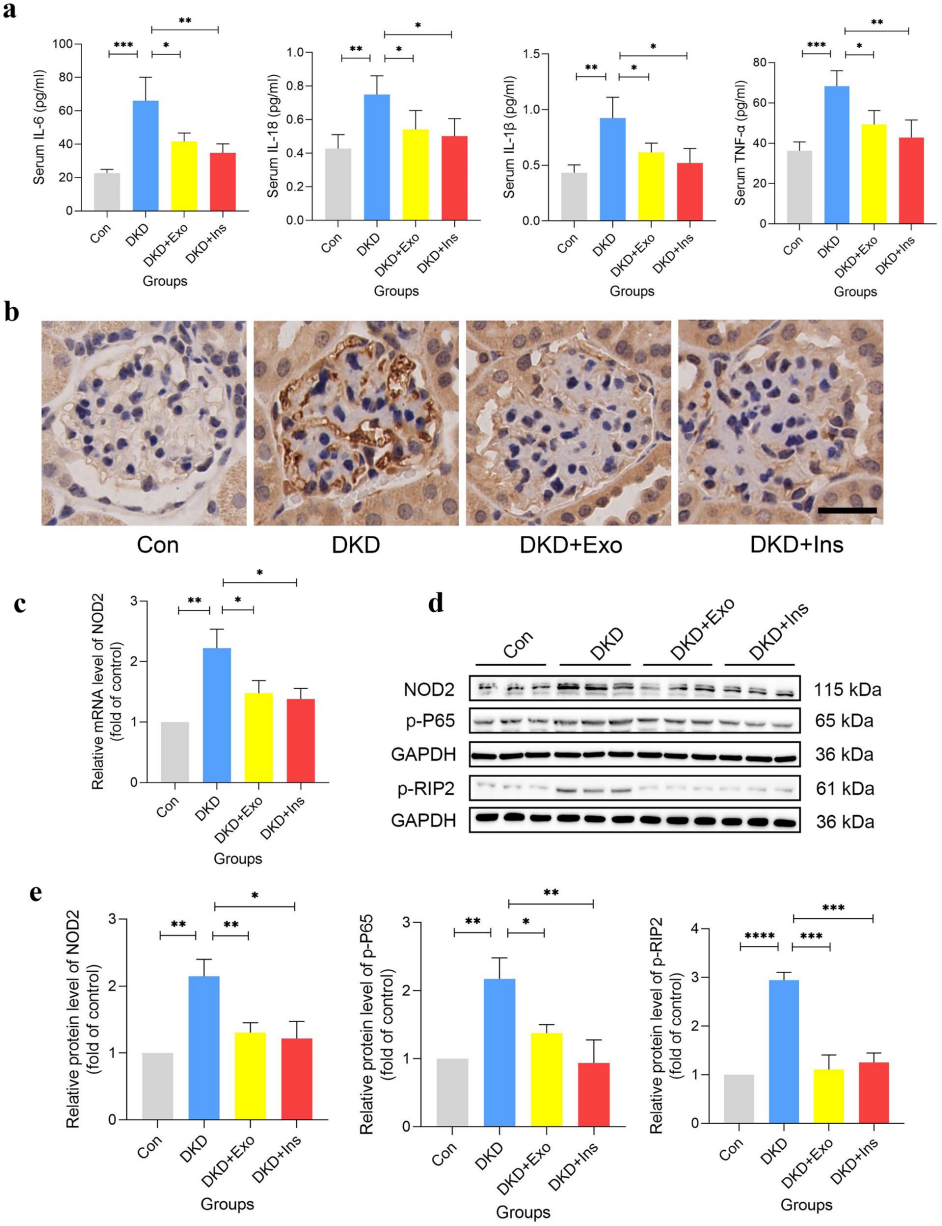
MSCs-Exo specifically inhibit NOD2 signaling activation in DKD mice. (a) Levels of proinflammatory factors (TNF-α, IL-6, IL-1β, and IL-18) expressionin in different groups of mice (*n* = 4). (b) Representative immunohistochemical images of NOD2 in four groups of kidneys (*n* = 4, bar = 20μm). (c) Relative mRNA level of NOD2 in four groups of kidneys (n = 3). (d) (e) Representative western blot gel documents and summarized data showing the protein levels of NOD2, p-P65 and p-RIP2 in four groups of kidneys (n = 3). Exo: HUC-MSCs derived exosomes; Ins: insulin; Con: db/m mice; DKD: db/db mice; DKD + Exo: db/db mice with Exo; DKD + Ins: db/db mice with insulin. Data are represented as mean ± SD, *p < 0.05; **p < 0.01; ***p < 0.001; ^****^p < 0.0001.

**Figure 8.**
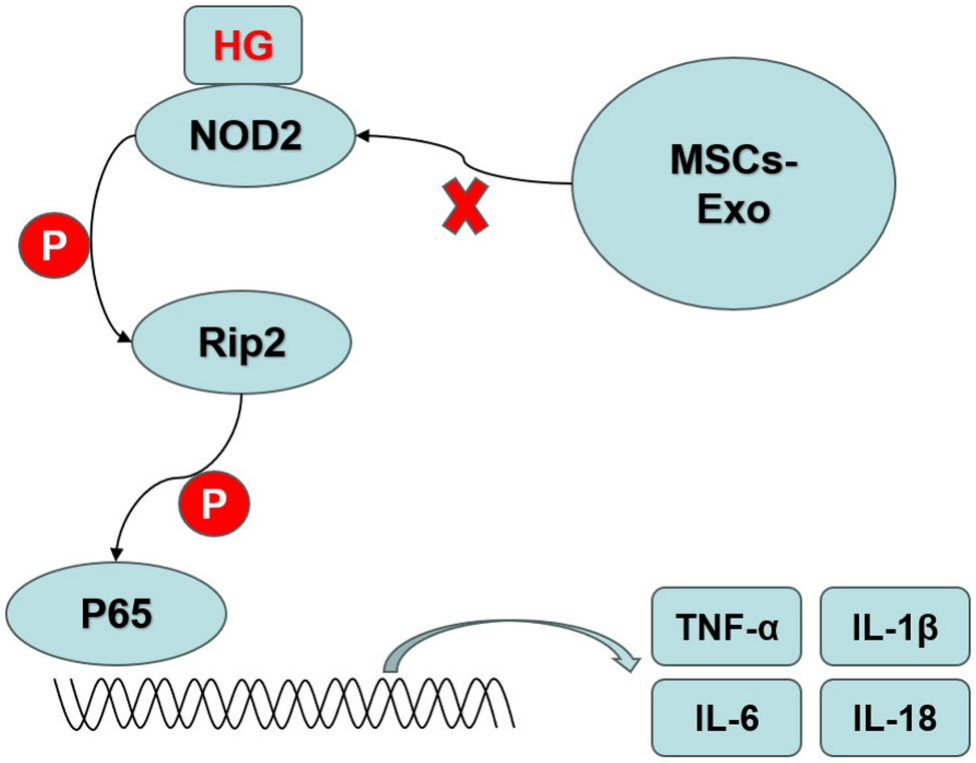
Graphical abstract. MSCs-Exo protected the podocytes from inflammation by mediating NOD2 pathway. P: phosphorylation.

Several studies revealed that NOD2 played important roles in the development of DKD. Therefore, to demonstrate the role of NOD2 signaling pathway in DKD mice, we detected the expression of NOD2 and its downstream proteins including p-P65, p-RIP2 as well as the inflammatory factors. As we expected, the expression of serum levels of the inflammatory factors TNF-α, IL-6, IL-1β, and IL-18 were significantly increased in DKD mice but decreased by MSCs-Exo and insulin treatment (Figure 7(a)). RT-qPCR, western blot and IHC indicated that the expression of NOD2 and its downstream proteins including p-P65, p-RIP2 was significantly increased in DKD groups and decreased in MSCs-Exo and insulin treatment groups (Figure 7(b–e)). Taken together, our findings suggest that NOD2 mediated inflammation activation may be mechanistically linked to DKD, and MSCs-Exo might perform their effect by inhibiting NOD2-mediated inflammation (Figure 8).

## Discussion

Podocytes as the key component of GBM, are highly specific terminally differentiated cell. As the terminally differentiated cells, podocytes are unable to replicate or significantly regenerate in adults,their injury may be the weakest common link and the primary insult leading to glomerulosclerosis and DKD [6,27]. More than 20% podocyte loss represents an irreversible step in DKD pathogenesis leading to glomerular scarring and development of ESRD [28]. In the state of diabetes, stimulation factors such as hyperglycemia and AGEs can cause podocyte injury [29]. Podocyte injury resulting in the destruction of GBM and proteinuria contributes to the pathogenesis of DKD [30]. Several research revealed that podocyte injury was associated with inflammation *in vitro* and *in vivo*. Although the exact pathogenesis of podocyte injury has not been fully elucidated, recent studies have found that innate immunity and the inflammation triggered by innate immunity, are closely related to the podocyte injury and function deficiency [31,32].

In recent years, many researchers demonstrated that the inflammation pathways play central roles in the progression of many diseases, including Crohn’s disease, atherosclerosis, Alzheimer’s diseases and DKD, and the targeted therapy to inflammatory molecules may provide new therapeutic strategies [33,34]. Toll-like receptors (TLRs) and Nod-like receptors (NLRs) are two major families of pattern recognition receptors. TLRs have been demonstrated to play a critical role in the innate immune system by activating proinflammatory signaling pathways in response to renal injury [35,36]. Our previous studies also demonstrated that Toll-like receptors, predominantly TLR2 and TLR4, recognize endogenous DAMPS, such as AGEs, HSP60, HSP70, and MCP-1 induce inflammatory responses *via* the nuclear factor-κB (NF-κB) signaling pathway in DKD; Moreover, we also proved that one member of NLRs: NLRP3 could mediated inflammation in podocytes under high glucose and DKD mice, resulting in the injury of podocytes and kidney, which is consistent with our previous study on the TLR signaling pathway.

NOD2 is the member of the NOD-like receptor family, plays an important role in innate immune response [37]. Since Du PC et al. firstly revealed that NOD2 was upregulated in the human diabetic kidney and diabetic mice and then demonstrated that podocyte injury was associated with inflammation mediated by NOD2 [7]. Increasing studies have revealed the contribution of NOD2 to innate immunity in DKD [10,38]. However, the exact molecular mechanisms and the association of NOD2 to the pathologies of DKD remains unknown. Muramyl dipeptide (MDP) is the ligand of NOD2, after binding with the ligand, NOD2 interact with downstream protein receptor interacting protein 2 (RIP2) and phosphorylate IκB then activated the transcription factor NF- κB, resulting the expression of inflammatory factors including TNF-α, IL-6, IL-1β, and IL-18, causing injuries to tissue and cells [39]. In this study, we firstly detected the expression of NOD2 in podocytes with or without high glucose *in vitro*, we found that both mRNA and protein level of NOD2 was significantly increased under high glucose. Similarly, we detected the expression of NOD2 *in vivo*, we found that no matter in HFD/STZ-induced DKD mice or db/db mice, the level of NOD2 was highly upregulated compared to the normal controls. Then we detected the NOD2 signaling pathway in podocytes and diabetic mice, our results showed that high glucose upregulated the expression of NOD2 as well as its downstream protein p-RIP2 and p-P65, resulting in the increase of inflammatory factors such as TNF-α, IL-6, IL-1β, and IL-18; *in vivo*, DKD mice had high expression of above proteins and inflammatory factors, our results were consistent with previous research that NOD2 is overexpressed in DKD.

Multiple studies have demonstrated that the administration of MSCs could prevent renal injury and promote renal recovery through a series of complex mechanisms [40]. Pan et al. found that injection of bone marrow-derived MSCs into DKD mice can repair damaged kidneys and pancreas, improving insulin resistance [41]. Li et al. found that MSCs can alleviate renal fibrosis in DKD by reducing the differentiation of myofibroblasts through paracrine secretion [42]. Bai et al. found that MSCs can alleviate DKD by reducing the release of inflammatory cytokines [43]. In 2013, we found that MSCs can improve glomerular fibrosis in DKD, improve renal function by inhibiting oxidative stress and macrophage infiltration [44]; Later, we found that MSCs can also inhibit the innate immune signaling pathways mediated by TLR2 and TLR4 in podocytes and DKD, reduce the expression of inflammatory factors, and significantly reduce physiological indicators including body weight, blood glucose, urinary albumin [20]. It is undeniable that MSCs have certain therapeutic effects on DKD both *in vivo* and *in vitro*. However, stem cell therapy still has certain limitations: whether MSCs from different donors or tissue sources have different proliferation rates and abilities; The expansion of MSCs *in vitro* can lead to stem cell aging and inevitably reduce their differentiation, migration, and regeneration abilities; In addition, MSCs transplantation also needs to consider its safety, immune rejection, and ethical approvement [45,46]. In recent years, more and more scholars have been convinced that MSCs are primarily mediated by the paracrine release of factors, including extracellular vesicles (EVs), composed of microvesicles and exosomes. Exosomes are one the key secretory products of MSCs, resembling the effect of parental MSCs. They can shuttle various proteins, messenger RNA (mRNA) and microRNAs (miRNAs) to modulate the activity of recipient cells. To date, MSC-derived exosomes (MSCs-Exo) have been successfully used in several preclinical models of chronic kidney disease including AKI, CKD, ischemia-reperfusion injury and DKD [47–49]. Moreover, we have proved that MSCs-Exo could target NLRP3 to inhibit the activation of the NLRP3 signaling pathway and decrease inflammation in podocytes and DKD [23]. Based on our previous findings we then hypothesized that MSCs-Exo might have a similar effect on NOD2 signaling. *In vitro*, we co-cultured podocytes with or without MSCs-Exo under high glucose, we found that MSCs-Exo could prevent podocytes from apoptosis, increase podocytes viability, inhibit the activation of NOD2 signaling, decrease inflammation. *In vivo*, we injected MSCs-Exo into DKD mice, we observed the same positive effects of exosomes. Although we have demonstrated that MSCs-Exo exert protective effects by mediating NOD2, our experiments still have limitations. NOD2 knockdown *in vitro* and *in vivo* would reinforce the NOD2 dependency in the protective effects of MSCs-Exo, however, due to the limited time and funds, it is hard for us to finish the supplement experiment of knockdown NOD2 in the current research. Besides, the exact mechanism about the activation of NOD2 signaling pathway *in vitro* and *in vivo* was not yet illuminated, therefore, in our next study, we need to clarify the specific mechanism of NOD2 activation and the exact mechanism of NOD2 inhibition by MSCs-Exo to strengthen our research through NOD2 knockout experiments including siRNA or shRNA.

In summary, our findings demonstrate that NOD2 is one of the critical components of a signal transduction pathway that links inflammation and injury in podocytes and DKD. MSCs-Exo targeting of NOD2-mediated signaling pathway may provide a new promising cell-free therapeutic strategy for DKD in the future.

## Data Availability

The data that support the findings of this study are available on reasonable request from the corresponding author.
